# Role played by MDSC in colitis-associated colorectal cancer and potential therapeutic strategies

**DOI:** 10.1007/s00432-024-05755-w

**Published:** 2024-05-08

**Authors:** Kang Wang, Yun Wang, Kai Yin

**Affiliations:** 1 Department of General Surgery, Affiliated Hospital of Jiangsu University, Jiangsu University, Jiefang Road No. 438, Zhenjiang, Jiangsu Province 212000 China; 2 Department of Dermatology, The First People’s Hospital of Changzhou, Juqian Street, Changzhou, Jiangsu Province 213003 China; 3 Department of General Surgery, Affiliated Hospital of Jiangsu University, Institute of Digestive Diseases, Jiangsu University, Jiefang Road No. 438, Zhenjiang, Jiangsu Province 212000 China

**Keywords:** MDSC, Colitis-associated colorectal cancer, CRC, Targeting strategies

## Abstract

Colitis-associated colorectal cancer has been a hot topic in public health issues worldwide. Numerous studies have demonstrated the significance of myeloid-derived suppressor cells (MDSCs) in the progression of this ailment, but the specific mechanism of their role in the transformation of inflammation to cancer is unclear, and potential therapies targeting MDSC are also unclear. This paper outlines the possible involvement of MDSC to the development of colitis-associated colorectal cancer. It also explores the immune and other relevant roles played by MDSC, and collates relevant targeted therapies against MDSC. In addition, current targeted therapies for colorectal cancer are analyzed and summarized.

## Introduction

Colorectal cancer (CRC) is the third most prevalent cancer worldwide and one of the leading causes of cancer-related deaths. With economic development and lifestyle changes, its incidence has been increasing year by year (Morgan et al. 2023), and studies have shown that the incidence of CRC has increased in people under 50 years of age in the past few decades (Spaander et al. 2023), with a shift towards diagnosis at a younger and more advanced stage (Siegel et al. 2023), as the population continues to age, the global cancer burden is expected to increase, making it a global public health problem that should not be underestimated. Previous studies have shown that people with inflammatory bowel disease (IBD) have an increased chance of developing colorectal cancer over time, the development of colorectal cancer, particularly colitis-associated colorectal cancer, is strongly associated with a cumulative inflammatory burden (Yvellez et al. 2021). Recent studies have shown that MDSCs in the immune system have a major impact on colorectal cancer progression, and together with many other immune cells, they jointly stimulate the proliferation of tumor cells, and participate in local angiogenesis and distal tumor metastasis.

In inflammatory bowel disease (IBD), intestinal inflammation and massive infiltration of bone marrow and lymphocytes are the main pathological features. It has been demonstrated that dendritic cells (DCs) and macrophages play a crucial role in controlling the development of pro-inflammatory lymphocytes, such as helper T cells and Th17 cells, within the intestines of individuals suffering from inflammatory bowel disease, and that pro-inflammatory lymphocytes further attract myeloid cells, including MDSC, into localized inflamed intestinal tissues. Currently, it has been found that MDSC infiltration is observed in animal models of CAC and patients with CAC, and these myeloid cells play a crucial role in facilitating the progression of IBD to CAC. Moreover, investigating the regulatory network of MDSCs in the development of CAC can help to explore new anti-tumor immunotherapeutic regimens for CAC targeting MDSCs, and enhancing the therapeutic effect of anti-CAC treatment is a novel approach. However, the mechanisms by which MDSCs control the progression of IBD to CAC remain largely unexplored. Here, we summaries the potential mechanisms of action of MDSC in the development of CAC from inflammatory bowel disease at this stage with other roles such as immune pathways, cytokines, and intestinal flora, and the potential ways in which MDSCs impact intestinal epithelial cells, resulting in the formation of low-grade and high-grade allopatric hyperplasia, and summarize the current therapeutic strategies for targeting MDSC-related CAC, as well as the research stages, existing findings and potential shortcomings of related studies, provide new insights into the specific ways in which MDSCs contribute to the progression of inflammatory bowel disease (IBD) to colorectal cancer (CAC) in the future, and also help to further explore the potential targets related to MDSCs for future CAC treatments, opening up new possibilities for effective therapeutic interventions.

## MDSC and colitis-associated colorectal cancer

MDSCs play a critical role in the progression of colorectal cancer associated with colitis. MDSC are a diverse group of immature cells that originate from myeloid cells. In the bone marrow, hematopoietic progenitor cells normally undergo a process of differentiation into myeloid progenitor cells. These myeloid progenitor cells then continue to differentiate into granulocyte–macrophage precursors, followed by further differentiation into monocyte/dendritic cell precursors as well as mature myeloid cells, etc., and travel to secondary lymphoid organs, where they undergo further differentiation into monocytes and neutrophils to carry out specialized tasks (Wu et al. 2022b). MDSC can be broadly classified into polymorphonuclear cells (PMN-MDSC) and monocytes (M-MDSC), which share phenotypic and morphological similarities with neutrophils and monocytes, respectively. The myeloid cell markers CD33 + , CD11b + , HLA-DR low/- and Lin- can be used to identify human MDSC, which are divided into two main subgroups, the M-MDSC (CD33 + CD11b + CD14 + CD15- HLA-DR low) subgroup and the PMN-MDSC (CD33 + CD11b + CD14- CD15 + HLA-DR-) subpopulation (Gabrilovich 2017). However, under conditions of chronic inflammation or tumor, due to the abnormal proliferation of bone marrow, a variety of pro-inflammatory factors may be produced, interfering with the normal maturation of myeloid cells and lead to an increase in the number of immature myeloid cells, and these heterogeneous populations, which have similar physical characteristics to monocytes or granulocytes but whose specific surface molecular signatures differ from those of monocytes or granulocytes are collectively referred to as MDSCs, whose main feature is the potent immune-suppressing function under pathological conditions such as inflammation or tumor. MDSCs can exert immunosuppressive effects through a variety of pathways and mechanisms, including the upregulation of nitric oxide synthase (iNOS), arginase-1 (ARG-1), reactive oxygen species (ROS), etc. to inhibit lymphocytes, and indirectly inhibiting the body’s immune response by suppressing regulatory T cells (Tregs). Recent research has shown a strong link between MDSC expression and the advancement of CAC during the transition from colitis to CAC using clinical specimens and mouse animal models, and MDSCs have become a key target in current oncology research due to their abundant presence in the tumor microenvironment (Chen et al. 2022a, b). MDSC infiltration is often seen at sites of inflammation in patients with chronic inflammatory diseases and tumor, relevant studies have shown that certain pro-inflammatory mediators present in the microenvironment, like IL-6, contribute to the promotion of MDSC accumulation in the pathological state of chronic inflammation and malignancy (Laws et al. 2023). Prostaglandin E2 (PGE2) is a significant lipid mediator that produced in the sustained inflammatory response, also further recruits MDSC, which in chronic inflammation further produce S100A8/A9 proteins, these calcium-binding proteins are mainly secreted by neutrophils and activated monocytes, and interestingly, these proteins in the inflammatory microenvironment in the inflammatory microenvironment further recruiting more MDSC causing further MDSC accumulation, the inflammatory microenvironment can also further increase the generation of reactive oxygen species (ROS) and pro-angiogenic factors like vascular endothelial growth factor (VEGF), which can contribute to MDSC accumulation and immunosuppressive activity, in addition, the tumor microenvironment and persistent inflammatory stimuli can also lead to an additional boost in the production of tumor necrosis factor alpha (TNF-α) by tumor cells, which also further recruits MDSC and enhances MDSC-associated immunosuppressive activity (Wang et al. 2023a, b, c, d, e). Multiple research studies have consistently shown that reducing MDSCs can effectively slow down tumor progression and lead to anti-tumor effects, and all these evidences suggest that MDSCs play unique and crucial roles in the progression of inflammatory bowel disease to CAC development (Krishnamoorthy et al. 2021; Liao et al. 2019; Wang et al. 2023e).

### Potential role of MDSC

The primary feature of MDSC is their capacity to inhibit the immune response. Each subtype of MDSC has different characteristics that affect their ability to regulate various components of the immune response. For example, PMN-MDSC primarily utilizes prostaglandin E2 (PGE2), arginase 1 and ROS for immunosuppression, whereas M-MDSC utilizes NO, IL-10, TGF-β, and PD-L1 for the same purpose (Youn et al. 2008).

#### Immune pathway related

##### STAT3 pathway

In the tumor microenvironment, MDSC are activated by multiple mechanisms. The crucial role is played by the transcription factor STAT3 (Zhang et al. 2024). STAT3 is an important oncogenic transcription factor, and phosphorylation of Bcl-2 regulates the expression of genes that prevent apoptosis, angiogenesis-related (e.g., VEGF), and some specific factors (e.g., S100A9), which are all relevant to colitis-associated colorectal cancer tumor invasion, metastasis and prognosis.

MDSC express receptors for S100A8 and S100A9, Wang et al. (2020) used CRISPR CAS9 to knock down specific immunosuppressive factors in a mouse tumor model and found that STAT3 has a significant role in controlling the differentiation of MDSCs. Additionally, STAT3 was found to increase the expression of S100A8/9 proteins, which promotes the clustering of MDSCs in the tumor microenvironment (TME). This clustering ultimately leads to the activation and multiplication of MDSCs (Wang et al. 2023a, b, c, d, e). Using single-cell cytokine profiling for immunoassay, G. Qin et al. found that MDSCs were significantly expressed in both clinical samples and mouse models of tumors, Additionally, they observed that specific factors like GM-CSF, G-CSF, IL-6, VEGF, etc., amplified the quantity of MDSCs in the tumor microenvironment and impeded their subsequent differentiation, and these factors further activated the JAK/STAT signaling pathway to stimulate myeloid cell production and promote MDSC expansion (Qin et al. 2023). Wu et al. found that the only inhibitory receptor in the Fcγ receptor family, FcγRIIB, was expressed in tumor-infiltrating MDSCs by constructing a mouse model of Fc γ receptor IIB receptor deficiency in the Immunoglobulin (Ig) Fc region, and using adoptive cell transfer, mRNA sequencing, and flow cytometry analysis to discover that the only inhibitory receptor in the Fcγ receptor family was responsible for upregulating the presence of tumor-infiltrating MDSCs. This receptor also played a role in promoting the production of MDSCs by hematopoietic progenitor cells through the Stat3 signaling pathway, thereby bolstering the immunosuppressive functions of MDSCs, resulting in tumor immune evasion (Wu et al. 2022a, b). In addition, there are also studies on multifunctional spore shell nanoparticles (CN) encapsulated on the surface of probiotics, and it was found that CN-encapsulated probiotics were successful in treating inflammatory bowel disease and preventing colorectal cancer. The activation of STAT3 was discovered to enhance the functionality of epithelial cells and prevent the death of intestinal epithelial cells (IECs). This, in turn, has an impact on the integrity of the intestinal barrier and the stability of the intestinal microenvironment, and that blocking the IL-6 -STAT3 signaling pathway through CN-encapsulated probiotics could prevent colitis-related colorectal cancer. Blocking the IL-6-STAT3 signaling pathway by CN-encapsulated probiotics prevented the prevention of colitis-associated colorectal cancer was achieved, which further confirmed the pathogenic role of STAT3 in colitis-associated colorectal cancer (Song et al. 2021).

##### NF-κB pathway

Studies have confirmed that IL-1β activates the NF-κB signaling pathway to promote MDSCs mobilization and proliferation. As a result, the progression of gastritis and gastric cancer is promoted (Tu et al. 2008). One of the functions of activating the NF-κB signaling pathway is to promote the mobilization of MDSCs, which in turn promotes colorectal tumorigenesis associated with ulcerative colitis. This study found that the glycoheterotrophic enzyme fructose 1,6-bisphosphatase 1 (FBP1) can directly interact with IκBα, thereby inhibiting NF-κB activation and suppressing colorectal tumorigenesis (Zhu et al. 2023). When VEGF is highly expressed in the TME, it can activate the NF-κB pathway. As a result of this activation, FLT3L expression is suppressed, which promotes the abnormal differentiation of myeloid progenitor cells into MDSCs. The NF-κB family member c-Rel was utilized in the study, and the use of a small-molecule inhibitor targeting c-Rel led to a notable reduction in the tumor-suppressing capabilities of MDSCs, ultimately resulting in the inhibition of tumor growth (Li et al. 2020). The regulation of apoptosis and proliferation of IECs is significantly influenced by the NF-κB signaling pathway. Furthermore, it plays a crucial role in preserving the integrity of the intestinal barrier and promoting the body’s defense against pathogens. The NF-κB signaling pathway is essential for preserving the integrity of the intestinal epithelium and ensuring a balanced immune response in the intestine, when NF-κB is deficient, this leads to the death of IECs, decreased production of antimicrobial peptides, and the movement of bacteria into the mucosa. These events contribute to the progression of inflammatory bowel cancer (Nenci et al. 2007). The close relationship between the NF-κB signaling pathway and the formation, recruitment, and activity of MDSCs is clearly evident, and is a key factor in colitis-associated colorectal cancer.

##### COX-2/PGE2 pathway

MDSC inhibitory activity is significantly influenced by prostaglandins, particularly prostaglandin E2 (PGE2). PGE2 has been linked to the promotion of tumor growth, angiogenesis, and the suppression of the immune system.

Relevant studies have shown that PGE2 inhibits immunity by recruiting MDSC, among others, and contributes to tumor angiogenesis. In the early stage of tumor formation, COX-2/PGE2 pathway can drive the inflammatory microenvironment, which promotes downstream signaling, and consequently, tumor progression in an irreversible direction. In the in vivo environment of colitis-associated colorectal cancer patients, overexpression of COX-2 leads to increased production of vascular growth factors. This, in turn, stimulates endothelial cell migration, leading to increased invasiveness and the spread of cancer cells is inhibited by reducing the Bcl-2 gene expression and decreasing apoptosis, etc. Thus, increased expression of COX-2 is associated with the development, progression and spread of colorectal cancer. CXCL1 is a pro-angiogenic CXCL1 is a pro-angiogenic chemokine, and PGE2 can induce CXCL1 expression, increase MDSC infiltration in colitis-associated colorectal cancer mouse models, promote in vivo tumor growth and increase tumor micro vessel formation. In recent years, there is strong evidence indicating that the prolonged use of NSAIDs significantly decreases the likelihood of developing colorectal cancer (CRC) (Friis et al. 2015), an interesting finding that has led to a reexamination of the "miracle drug" aspirin and its target cyclooxygenase (COX), COX is responsible for metabolizing arachidonic acid, released from membrane phospholipids by phospholipase A2 (PLA2), into prostaglandin H2 (PGH2) via specific isoenzymes and TXX2, an enzyme that is a key component of prostaglandin H2 (PGH2). COX has the ability to break down arachidonic acid, which is released from membrane phospholipids by phospholipase A2 (PLA2), and convert it into prostaglandin H2 (PGH2), which is converted by specific isomerase and TXA synthase into different prostaglandins (such as PGE2, PGD2, PGF2α, PGI2, etc.) and TXA2, and COX has two isozymes, COX1 and COX2, with COX-1 being responsible for the maintenance of tissue homeostasis during regular physiological conditions. COX-1 is primarily responsible for maintaining the necessary basal levels of prostaglandins for tissue balance in normal physiological situations. On the other hand, COX-2 is predominantly found in inflammatory cells (Ye et al. 2020). Recent research has demonstrated that elevated levels of COX-2 in colon tumor cells lead to an increased production of PGH2 from arachidonic acid, and therefore the expression of PGE2 is increased in the microenvironment. PGE2 can enhance the progression of colorectal cancer by inducing apoptosis, promoting cell proliferation, stimulating angiogenesis, and facilitating metastasis (Karpisheh et al. 2019). The expression of MYO10 (signaling axis) is decreased when COX-2 is knocked down, resulting in a decrease in the migratory and invasive capabilities of colon cancer tumor cells (Liu et al. 2023a, b). At the same time, COX-2 inhibitors, resist epithelial-mesenchymal transition (EMT) alterations in CRC cell lines (This is also a key process in cancer cell metastasis, where epithelial cells undergo a transformation from their original characteristics to mesenchymal characteristics, resulting in enhanced motility and migratory abilities). It has also been shown that co-targeting COX2 with BRAF + EGFR durably inhibits tumor growth capacity in patient-derived tumor xenograft models (Ruiz-Saenz et al. 2023). COX2 inhibition represents a strategy that could overcome associated CRC treatment resistance. Currently, a variety of COX-2 small molecule inhibitors, such as rofecoxib, have entered the phase III clinic (NCT00031863), and other small molecule inhibitors, such as imrecoxib (ChiCTR2100051644), lumiracoxib (NCT00170898), and meloxicam (NCT01886872), have also entered the clinic and have been successfully marketed. NCT01886872) have also entered the clinic and been successfully marketed. It is believed that with the development of COX-2 inhibitors with higher safety and specificity in the future, it will be more helpful in preventing and controlling the development of tumors.

#### Cytokine-related

New research has indicated that specific inflammatory cytokines and chemokines have the ability to promote the proliferation of MDSCs. On one side, they are withholding crucial amino acids from T cells, induce oxidative stress, and are involved in regulating the functions of Tregs and T helper (Th)17 cells, etc. On the other hand, They stimulate MDSCs to release reactive oxygen radicals (ROS), inducible nitric oxide synthase (iNOS), and arginase 1 (Arg-1), which suppress T cell activity and promote T cell apoptosis (Ma et al. 2020). At the same time, an imbalance between pro-inflammatory and anti-inflammatory cytokines can fuel the progression of IBD, leading to the development of colitis-associated colorectal cancer.

IL-10: A cytokine known to suppress inflammation. Chronic inflammation in ulcerative colitis contributes to the accumulation of high levels of MDSCs in the colon, and in turn, high levels of MDSCs produce higher levels of IL-10, but the function of IL-10 is altered in this environment IL-10 instead activates STAT3, which results in increased expression of two genes, DNMT1 and DNMT3b-, contributing to the silencing of a tumor suppressor (Ibrahim et al. 2018). Leading to possible IEC heteroplasia, driving a higher incidence of inflammatory bowel cancer.

IL-6: The progression of colitis-associated colorectal cancer was also significantly affected by IL-6. It has the ability to activate Janus kinase (JAK) and induce phosphorylation of the transcription factor STAT3 downstream, thus promoting the development of cancer. Apart from the JAK/STAT3 pathway, IL-6 can also exacerbate the intestinal inflammatory response in patients and promote colitis-associated colorectal cancer by regulation of Th17 and Treg cell proliferation and function (Wang et al. 2023a, b, c, d, e). IL-6 produced by MDSCs can hinder the development and activity of CD4( +) T cells, ultimately promoting tumor development (Tsukamoto et al. 2013). It has been shown that blocking IL-6 enhances the therapeutic effect of immunotherapy by inducing and recruiting higher levels of CD4 + /CD8 + effector T cell production and recruitment in the tumor microenvironment (Hailemichael et al. 2022).

TGF-β: It can exert its anticancer effects through antiproliferative, pro-apoptotic and inhibiting the production of inflammatory factors with pro-tumor activity. However, TGF-β also modulates the immunosuppressive effects of MDSCs and hinders the ability of immune cells to fight against tumors, and also promotes epithelial-mesenchymal transition (EMT), thus accelerating tumor metastasis (Yang et al. 2008).

TNF-a: It is an important pro-inflammatory factor that plays a crucial role in the advancement and growth of colorectal cancer linked to colitis. This inflammatory mechanism is primarily responsible for the sustained activation of the NF-κB signaling pathway. It can also stimulate colitis-associated colorectal cancer production by damaging DNA. It has been shown that TNF-α indirectly mediates the effects of ROS on the stem cell microenvironment and plays an important role in epithelial cells (Hsu et al. 2022). Blocking TNF can attenuate colorectal cancer by altering the composition and activity of the microbiota (Yang et al. 2020).

#### Other relevant avenues

In the corresponding environment, MDSC release reactive oxygen radicals (ROS), etc. The large amount of reactive oxygen ROS etc. produced can cause oxidative stress. Studies have shown that oxidative stress induces the misvocalization of nuclear RNA or the DNA-binding protein TDP-43, which further leads to the overaccumulation of the R-loop (i.e., DNA in the form of an RNA heterozygous strand structure formed by DNA and RNA), which in turn triggers DNA damage and instability in the genome, resulting in the over-activation of the key enzyme for DNA repair, PARP1. This further trigger depletion of the coenzyme NAD + and ATP deficiency, which promotes the onset of mitochondria-dependent necrotic apoptosis in intestinal epithelial cells, which in turn drives the onset of spontaneous intestinal inflammation (Yang et al. 2024). In addition, excess ROS can cause damage to intestinal cells. This damage occurs through a variety of mechanisms, including induction of DNA mutations, impairment of protein function, alteration of epithelial permeability, and disruption of the intestinal epithelial barrier. These effects ultimately lead to cancer development and the proliferation of tumor cells (Wang et al. 2023a, b, c, d, e; Zhang et al. 2023). Thus, inflammation-induced oxidative stress is clearly also important in the progression of colitis-associated colorectal cancer.

Several studies have shown that the metabolism of intestinal flora can also be closely related to the progression of colitis-associated colorectal cancer. Associated microbiota can induce aggregation of MDSCs and pro-inflammatory functions. For example, the presence of commensal Gram-negative gut bacteria can lead to the accumulation of MDSCs (Zhang et al. 2020). FadA adhesin produced by Clostridium nucleatum binds to E-calmodulin to produce pro-inflammatory factors and activates the TLR4/NF-κB signaling path way via lipopolysaccharide LPS to promote the recruitment of MDSCs to the infection site, this recruitment activates the Wnt/β-catenin signaling pathway, which further modulates the bacterial adhesion and invasion into the epithelial cells, ultimately, this process promotes the proliferation of colorectal cancer cells that Promotion of colorectal cancer (Yang et al. 2024). Enterotoxin-producing bacterium Enterobacteriaceae fragilis (ETBF) induces Th17 recruitment and inhibits T-cell proliferation, contributing to a pro-inflammatory milieu that favors the production and differentiation of pre-tumorigenic monocyte-derived cells (MDSCs), as a consequence, the chances of developing colorectal cancer may increase (Thiele Orberg et al. 2017).

**Fig.1:**
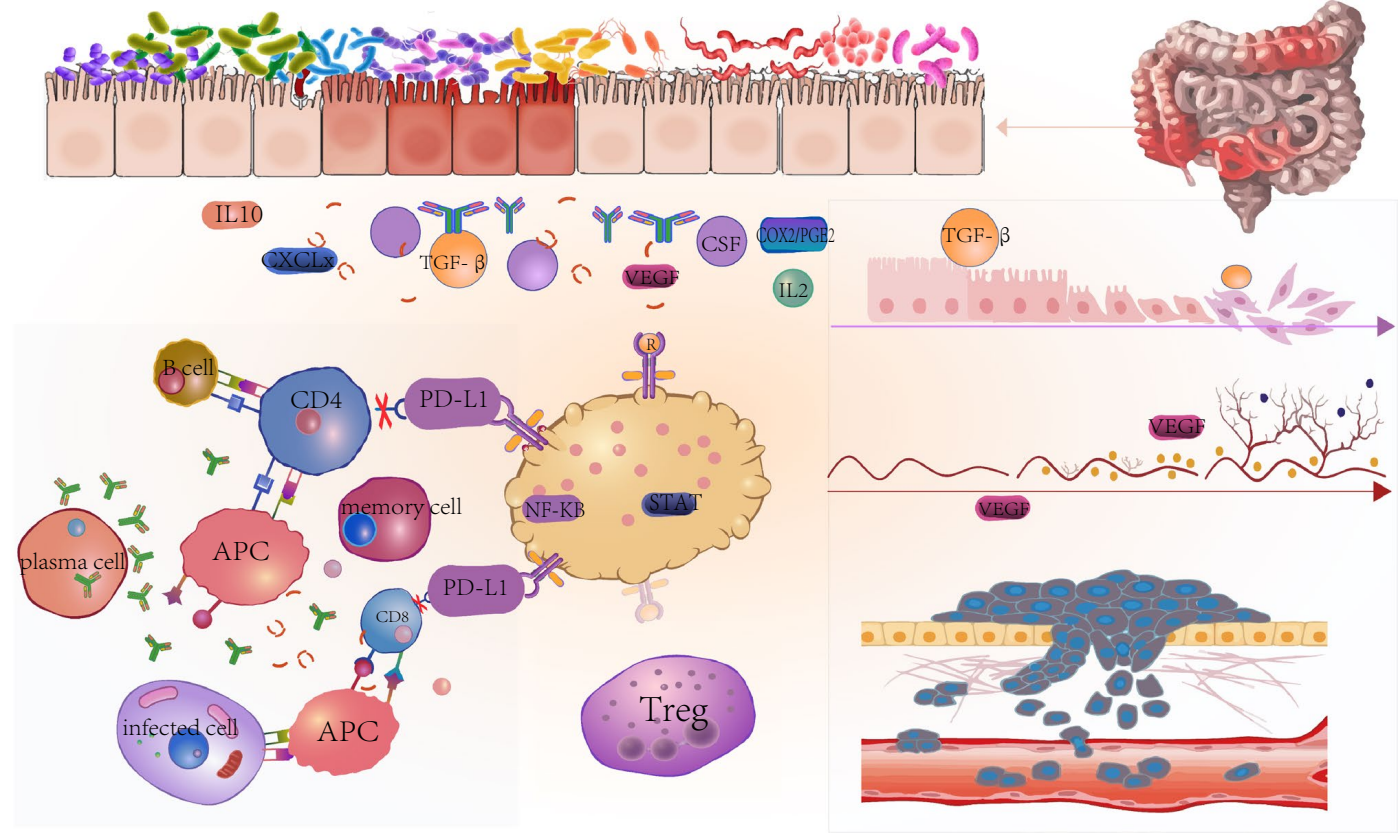
MDSC exerts relevant immunosuppressive functions in the inflammatory microenvironment and influences tumor development. Associated chemokines (CSF, VEGF, CXCLx, etc.) recruit MDSC in the inflammatory microenvironment; MDSC play an immunosuppressive role by inhibiting T-cell-associated functions; MDSC promote the involvement of Treg cells in the associated suppression of anti-tumor immune responses; MDSC promote epithelial-mesenchymal transition (EMT), tumor angiogenesis, and enhancement of tumor cell stemness

Figure 1 illustrates the involvement of MDSC in colitis-associated colorectal cancer. However, the specific role of MDSC in the advancement of colitis-associated colorectal cancer is not fully understood, and more research is necessary to gain a comprehensive understanding of how MDSC contributes to the formation of inflammatory bowel cancer.

### Effects of MDSC on intestinal epithelial cells

A sustained and persistent inflammatory response is critical to the development of colorectal cancer associated with colitis. This inflammatory response plays a role in every stage of tumor formation and development. Colitis significantly increases the risk of colorectal cancer due to the long-term accumulation of inflammation. Chronic inflammation and increased epithelial cell renewal will lead to the formation of both low-grade and high-grade heterogeneous proliferation of intestinal epithelial cells, which may promote the further transformation of IBD to colitis-associated colorectal cancer, and the poor prognosis of patients who progress from inflammatory bowel disease to colorectal cancer has a higher mortality rate. Recent studies have shown that patients with inflammatory bowel disease, MDSC promotes the proliferation of intestinal epithelial cells, triggers heteroplasia of intestinal epithelial cells, and enhances the ability of tumor cells to maintain stem cell properties, thus contributing to irreversible cancerous transformation of intestinal epithelial cells.

#### Promotes proliferation of IECs

Relevant studies have shown that under conditions of inflammatory bowel disease, MDSC-derived IL-6 activates STAT3 and stimulates intestinal epithelial cell IEC hyperproliferation. Invasiveness, proliferation and stemness of human colon cancer cells are closely related to Stat3 activation (Liu et al. 2022). Furthermore, it has been shown that elimination of Stat3 from epithelial cells greatly hinders the development of colitis-associated colorectal cancer. This is achieved by inhibiting cell proliferation and promoting apoptosis. It has also been shown that removal of cyclic guanosine-adenylate synthase (cGAS) promotes the recruitment and activation of MDSCs in the colon, and proliferation of intestinal epithelial cells and increased permeability of the intestinal barrier. It may promote colonic inflammation and cancer development (Hu et al. 2021).

#### Elicits heterogeneous hyperplasia in the IECs

MDSC promote tumor progression by promoting chronic inflammation, facilitating angiogenesis, and establishing a tumor microenvironment that suppresses the immune system. It has been shown that PAR2 deficiency in MDSC directly enhances its immunosuppressive activity by promoting STAT3-mediated reactive oxygen species production, contributing to IEC heteroproliferation (Ke et al. 2020). In the microenvironment of inflammatory bowel disease, MDSC-derived ROS may cause IEC heteroproliferation by damaging IEC DNA, inducing DNA alterations in epithelial cells and inhibiting their repair. Under inflammatory conditions, IL-6-activated STAT3 also plays an important role in IEC heteroplasia (Grivennikov et al. 2009).

#### Enhancement of tumor cell stemness

In an inflammatory environment, MDSCs can also promote colitis-associated colorectal cancer progression by promoting stemness in colon cancer cells. There have been reports indicating that netrin-1 inhibits PMN-MDSC recruitment and promotes tumor cell stemness, which is closely related to drug resistance and cancer recurrence after chemotherapy or immunotherapy (Ducarouge et al. 2023). MDSC-associated PGE2 induces iPSC in mice to acquire the characteristics of cancer stem cells (CSCs) through the PI3K/Akt axis. Thus, it is clear that PGE2 also contributes to the promotion of cancer cell stemness that leads to colitis-associated colorectal cancer (Minematsu et al. 2022).

## Targeting MDSC-related therapeutic strategies

MDSCs consistently recruited in the tumor microenvironment have the ability to inhibit T cell proliferation and impair their function. Therefore, targeting MDSCs is a potential strategy for corresponding tumor therapy. We summarized the current relevant studies targeting MDSC therapy as shown in Table 1.

**Table 1:** Current targeted therapeutic strategies for MDSC

Drug type	Impact on MDSC	Drug	Research phase	Tumor type	References
VEGF inhibitor	Inhibits MDSC recruitment	Bevacizumab	Phase 2 clinical trial NCT01730950	Glioblastoma	Tsien et al. (2023)
ENTPD2 inhibitor	Stops MDSC accumulation	POM-1	Pre-clinical studies	Hepatocellular carcinoma	Chiu et al. (2017)
HIF-1α inhibitor	Induces MDSC differentiation	PX478	Phase 1 clinical trial NCT 00522652	Solid tumor	Lee and Kim (2011)
LXR agonist	Depletes MDSC	RGX-104	Phase 1 clinical trial NCT02922764	Lung cancer lymphoma	(Wan et al. 2020)
S100A8/A9 inhibitor	Stops MDSC accumulation	Tasquinimod	Phase 3 clinical trial NCT01234311	Prostate cancer	Mehta and Armstrong (2016)
CCR2 inhibitor	Inhibits MDSC recruitment	PF-04136309	Phase 2 clinical trial NCT01413022	Pancreatic	Nywening et al. (2016)
CXCR2 inhibitor	Stops MDSC accumulation	SX-682	Phase 2 clinical trial NCT05604560	Pancreatic	Liu et al. (2023a, b)
CCR5 inhibitor	Inhibits MDSC recruitment	Maraviroc, Pembrolizumab	Phase 1 clinical trial NCT03274804	Colorectal cancer	Haag et al. (2022)
CSF1-R inhibitor	Inhibits MDSC recruitment	ARRY-382	Phase 1 clinical trial NCT02880371	Solid tumor	Johnson et al. (2022)
Ant pyrimidine drug	Depletes MDSC	5-FU	Phase 3 clinical trial NCT02055560	Colorectal cancer	Akdeniz et al. (2021)
Pyrimidine antimetabolite	Stops MDSC accumulation	Gemcitabine	Phase 3 clinical trial NCT04003636	Biliary tract cancers	Kelley et al. (2023)
Third-generation platinum-based cytotoxic drugs	Depletes MDSC	Oxaliplatin	Phase 3 clinical trial NCT00275210	Colorectal cancer	Webster-Clark et al. (2022)
Diterpene alkaloids	Depletes MDSC	Paclitaxel	Phase 3 clinical trial NCT01839773	Gastric cancer	Kang et al. (2018)
Tyrosine kinase inhibitor	Stops MDSC accumulation	Sorafenib	Phase 3 clinical trial NCT00492752	Hepatocellular carcinoma	Cheng et al. (2009)
Biguanide	Inhibits hif-1α expression, induces MDSC differentiation	Metformin	Phase 2 clinical trial ChiCTR-ICR-15005940	Esophageal squamous carcinoma	Hayter et al. (2000)
VitA metabolic intermediate	Induces MDSC differentiation	ATRA	Phase 2 clinical trial NCT03200847	Metastatic melanoma	Tobin et al. (2023)
STAT3 inhibitor	Induces MDSC differentiation	Danvatirsen	Phase 1 clinical trial NCT01563302	DLBCL	Reilley et al. (2018)
TLR7/8 agonist	Stops MDSC accumulation	Resiquimoid (R848)	Pre-clinical studies	PDAC	Michaelis et al. (2019)
HDACinhibitor	Inhibits MDSC functional activity	Entinostat	Phase 2 clinical trial NCT01928576	Solid tumors	Luke et al. (2023)
PDE5 inhibitor	Inhibits MDSC functional activity	Tadalafil	Phase 3 clinical trial NCT00843635	Head and neck squamous cell carcinoma	Weed et al. (2015)
COX2 inhibitor	Inhibits MDSC functional activity	Celecoxib	Pre-clinical studies	Oral squamous cell carcinoma	Mabrouk et al. (2023)

*5-FU* 5-Fluorouracil, *ATRA* All-trans-retinoic acid, *DLBCL* large B-cell lymphoma, *PDAC* pancreatic ductal adenocarcinoma, *HDAC* Histone deacetylase

### Depletes MDSC

Chemotherapeutic agents such as gemcitabine, cyclic tetrazolamide (CTX), 5-fluorouracil, oxaliplatin, paclitaxel, and others have the potential to improve the effectiveness of immunotherapy in combating tumors. They achieve this by targeting the immunosuppressive cells present in the tumor microenvironment (Gürlevik et al. 2016). Gemcitabine, as a nucleoside analogue, is now widely used in the first-line treatment of various solid tumors. Gemcitabine can directly kill tumor cells and down reduce the quantity and activity of MDSCs, and enhance T-cell-mediated immune responses against tumors (Jiang et al. 2023). Relevant studies have shown that chemotherapeutic agents such as CTX can decrease the expression level of multidrug and toxin extrusion transporter protein ABCB1 in Treg cells, thereby depleting Treg and MDSC cells and inhibiting tumor growth (Galluzzi et al. 2020). Chemotherapeutic drugs such as fluorouracil (5-FU) has been recognized as an important drug for colorectal cancer since it was applied in the clinic in 1957. It can block the synthesis of the nucleoside thymidine, which is essential for DNA replication, and the relevant studies have found that MDSCs in several mouse tumor models display lower levels of the enzyme targeted by 5-FU. Moreover, cells with reduced thymidine synthase expression are highly vulnerable to cell death caused by 5-FU. Due to their low expression of thymidylate synthase, cells are highly susceptible to cell death induced by 5-FU. As a result, 5-FU can selectively eliminate MDSCs by inducing apoptosis, and the administration of 5-FU treatment significantly reduces tumor growth rate in mouse models and achieves therapeutic efficacy (Kim et al. 2021). A randomized controlled trial conducted on colon cancer patients in phase II has also shown that 5FU can effectively combat tumors by specifically targeting and eliminating MDSC. Studies conducted both in vitro and in vivo have demonstrated that 5FU has strong cytotoxicity against MDSC, thereby mediating immune tolerance. The 5FU-induced reduction in MDSC also further enhances the production of interferon-Y (IFN-Y) by CD8( +) T cells, which contributes to the promotion of anti-tumor responses (Wang et al. 2023a, b, c, d, e).

Tyrosine kinase inhibitors such as sunitinib can directly target the amplification signaling pathway of MDSC to achieve the purpose of MDSC depletion. Sunitinib is a targeted therapeutic drug that can inhibit the activity of multiple receptor tyrosine kinases, and relevant clinical studies have demonstrated that sunitinib inhibits the growth and spread of cancer cells, showing obvious anti-tumor activity and safety (Heinrich et al. 2024; Vallilas et al. 2021). A recent study reported that two multi-targeted tyrosine kinase inhibitors, carbonatitic or celecoxib, were able to reduce MDSC levels in a mouse model of squamous cell carcinoma (Huang et al. 2020). Additionally, there are studies that have assessed the effectiveness and safety of combining the tyrosine kinase inhibitor cabozantinib with a PD-L1 inhibitor in patients with advanced colorectal cancer within a specific colorectal cancer population. The results demonstrated that this treatment plan demonstrated significant effectiveness in fighting tumors and had tolerable side effects in patients with advanced colorectal cancer that did not respond to previous treatments (Saeed et al. 2024).

Depleting MDSCs continues to be a crucial approach for enhancing anti-tumor immunity, and as we uncover more MDSC-related targets, this strategy will become even more significant in the future, it will also surely provide new ideas for future therapeutic options.

### Induces MDSC differentiation

To successfully decrease the quantity of MDSC in mouse tumor models and cancer patients, we can also achieve our desired effect by inducing differentiation of immature myeloid cells.

Vitamins such as vitamin D and vitamin E can induce MDSC cell differentiation. Relevant studies have shown that 1,25-dihydroxyvitamin D3 plays a crucial role in controlling cell growth and differentiation, and recent research has demonstrated that 1,25-dihydroxyvitamin D3 has the ability to transform normal human myeloid cells into macrophages and monocytes, vitamin E enhances the immune response by reducing the production of ROS and NO and reducing the presence of immature MDSCs (O'Mahony et al. 2023). ATRA, also known as trans-retinoic acid, is a substance derived from vitamin A. It is used in the production of myeloid cells. The transformation of MDSCs into fully developed myeloid cells is significantly influenced by it. Several studies have demonstrated the ability of ATRA to activate the ERK1/2 kinase pathway and then upregulate glutathione, further scavenging ROS, resulting in a decrease in the amount of ROS in MDSCs. After administering ATRA treatment, the number of MDSCs in a mouse model of tumor showed a significant decrease, and MDSCs were stimulated to differentiate into dendritic cells and macrophages, which transformed their immune-suppressing function into an immunogenic one, greatly improving the anti-tumor effect. greatly improved the anti-tumor effect (Hengesbach and Hoag 2004). 24 patients with advanced melanoma participated in a phase 2 clinical trial (NCT03200847) to evaluate the effectiveness of combining ATRA and Pembrolizumab, it was demonstrated that the ATRA combination therapy was well-tolerated and safe in patients with melanoma. Additionally, it effectively decreased the proportion of MDSCs while increasing the proportion of myeloid cells, and that the majority of the patients achieved significant symptomatic relief. In other words, patients treated with ATRA had reduced levels of MDSCs and promoted MDSC differentiation, and this alteration in differentiation subsequently decreased the immunosuppressive effects of MDSCs, translating into enhanced immunotherapy efficacy and safe and effective treatment (Olson and Luke 2023).

Also some researchers have found in mouse tumor models that diosgenin not only triggers apoptosis in MDSCs, but also promotes their transformation into M1-like macrophages while reducing the number of M-MDSC cells. This discovery weakens the development of colitis-associated colon cancer, making it a promising natural option for effectively preventing CAC. We believe that in the future, we can explore more relevant drugs for inducing MDSC differentiation, which can help the treatment of CAC (Xun et al. 2023).

### Inhibits MDSC recruitment and migration

Under pathological conditions such as inflammation, tumors, etc., this leads to the recruitment and migration of MDSCs, which then exert their immunosuppressive function. Therefore, there are many studies to stop this process.

VEGF inhibitor vascular endothelial growth factor (VEGF) is a distinctive growth factor that specifically targets vascular endothelial cells, stimulating their growth and enhancing their functionality. It is essential for increasing vascular permeability, breaking down the extracellular matrix, supporting endothelial cell motility and growth, and stimulating the growth of fresh blood vessels via the process of angiogenesis. Tumor cells are the main source of VEGF and play a role in TME. VEGF, in turn, Stimulates the growth of fresh blood vessels (angiogenesis) and induces MDSCs to enter the tumor. To address this problem, monoclonal antibodies (mAb) have been developed that specifically target VEGF. These mAb have shown promising results in inhibiting tumor growth in mouse cancer models and in human patients (Shojaei et al. 2007). Preclinical and clinical studies have shown that bevacizumab, a recombinant human monoclonal antibody targeting vascular endothelial growth factor, is effective in reducing intratumorally MDSCs. During a Phase 2 clinical trial (NCT01730950), the utilization of bevacizumab demonstrated a significant improvement in progression-free survival (PFS) in patients with recurrent glioblastoma (GBM). This treatment effectively reduced the occurrence of MDSC and prevented MDSC recruitment and movement, resulting in clinically significant efficacy. A trial has shown a reduction in circulating MDSCs after combination therapy with bevacizumab and 5-fluorouracil, oxaliplatin in patients with colorectal cancer (Limagne et al. 2016a, b). Perhaps we can explore more relevant combination therapy options in the near future.

HIF-1α inhibitor: The increased expression of HIF-1α in the hypoxic environment of TME can lead to an increase in glycolytic enzymes and lactate transporter proteins in MDSCs as a way to promote MDSC differentiation and proliferation. Moreover, HIF-1α can also improve mitochondrial respiratory function through the PI3K/AKT and JAK2/STAT3 pathways, as well as attenuate cellular oxidative stress and reduce ROS production, thus alleviating MDSC cell injury. Meanwhile, the acidic environment formed by hypoxia promotes MDSC proliferation and enhances the inhibitory function of MDSC on T cells through HIF-1α. HIF1α hypoxia-inducible factor. Stable expression under hypoxic conditions. Plays an important role in MDSC accumulation. Therefore, MDSC recruitment in TME can be reduced by HIF1α inhibitors. Against hypoxia, a phase 1/2 clinical study (NCT01522872) involving TH-302 (efaproxiral), a hypoxia-activated prodrug, demonstrated extended survival in patients with myeloma, combating the effects of hypoxia (Laubach et al. 2019). In mouse kidney and mammary tumor models, researchers found that a nano-enzyme called Zr-CeO exhibited promising results in diminishing the recruitment of MDSCs, thereby enhancing the efficacy of PD-1 inhibitors in combating tumors (Mo et al. 2023).

Calcium-binding proteins S100A8 and S100A9, it plays a crucial role in the accumulation of MDSCs. Reduction of S100A8/A9 reduces MDSC accumulation in several mouse tumor models. Tiquinamide has been proven to decrease the infiltration and accumulation of MDSCs, with S100A9 as one of the targets. In a phase II clinical trial, results showed that administration of guanosine amide was effective in reducing the infiltration and accumulation of MDSCs, of which S100A9 is a specific target. This significantly slowed disease progression in metastatic CRPC. Furthermore, tiquinamide demonstrated enhanced progression-free survival rates in metastatic refractory prostate cancer (mCRPC) (Mehta and Armstrong 2016). The results indicate that the aggregation of MDSCs is significantly influenced by the involvement of S100A8/A9.

Chemokine receptors, it plays a key role in directing MDSCs to the tumor site. MDSCs are mainly identified by their expression of the chemokine receptor CCR2 and are attracted to tumors that produce the chemokines CCL2 and CCL5.

Research has demonstrated that blocking the CCL2/CCR2 axis with CCR2 antagonists, either on their own or in conjunction with other compounds, reduces the presence of MDSC in tumors and enhances tumor condition in preclinical mouse models (Nywening et al. 2016). The CCR2 inhibitor PF-04136309 has been shown to improve the survival of pancreatic cancer patients in combination with FOLFIRINOX, improves the chances of survival for individuals diagnosed with pancreatic cancer, demonstrating an improved antitumor response (NCT01413022).

In conclusion, these studies suggest that drugs targeting CCR2 can effectively decrease the levels of MDSCs and enhance the survival rates of individuals with cancer.

The small molecule inhibitor SX-682, which targets CXCR1 and CXCR2, effectively reduces the accumulation of tumor-infiltrating myeloid-derived suppressor cells (MDSCs). When used with checkpoint inhibitors, it has demonstrated promising results in improving the body’s immune response against tumors.

Studies have indicated that the combination of the CCR5 antagonist maraviroc with pembrolizumab is feasible in metastatic colorectal cancer (NCT03274804) (Liu et al. 2023a, b). It is uncertain whether these drugs will be successful in reducing the buildup of MDSCs and enhancing the immune system’s ability to fight tumors.

Targeting CSF1-R is also a method to block the migration of MDSCs to tumor locations. Binding of CSF1-R to ligand CSF1 stimulates the development and proliferation of myeloid cells. Studies performed in a mouse tumor model demonstrated that blockade of CSF-1R using a specific inhibitor (BLZ945) reduced the aggregation of MDSCs and shrunk tumor size. This highlights the important role of CSF-1R signaling in recruiting MDSCs (Mao et al. 2016).

Currently, a phase I clinical trial (NCT02880371) is in progress to assess the safety and initial effectiveness of the colony-stimulating factor-1 receptor-specific inhibitor ARRY-382 (PF-07265804). The trial is designed to evaluate the combination of ARRY-382 and pembrolizumab in advanced solid tumors (Johnson et al. 2022).

### Modulates the immunosuppressive function of MDSC

Modulating the immunosuppressive function of MDSCs has been utilized as a therapeutic approach to enhance the function of T cells and other immune cells.

The researchers found that by blocking iron death in the mouse model, they were able to eliminate the inhibitory effect of PMN-MDSC on T cells. This led to a significant reduction in tumor growth. Moreover, the use of iron death inhibitors combined with anti-PD1 treatment exerted a better anti-tumor therapeutic effect (Kim et al. 2022). M-MDSC induced an increase in adenosine levels, and depletion of adenosine in TME using polyethylene glycolate adenosine deaminase (PEG-ADA) improved the efficacy of immune checkpoint inhibitor (ICI) therapy in mouse studies. This provides new ideas for overcoming resistance to ICI in cancer patients (Sarkar et al. 2023).

As previously described, the accumulation and growth of MDSCs in tumors are greatly affected by the STAT family of transcription factors. Among these factors, STAT3 plays a particularly vital role in this mechanism.

The STAT3 inhibitor Napabucasin significantly increased the survival of melanoma-bearing mice. This inhibitor successfully eliminated the immunosuppressive capacity of mouse MDSC and human M-MDSC (Bitsch et al. 2022). Experimental evidence from a Phase 1b clinical trial (NCT01563302) demonstrated that the STAT3 inhibitor AZD9150 (Danvatirsen) was able to reduce granulocyte MDSC levels. In addition, Danvatirsen alone or in combination with checkpoint inhibitors has shown encouraging results in reducing PMN-MDSC levels in diffuse B-cell lymphoma. Combination therapy with Danvatirsen and the anti-PDL1 monoclonal antibody Durvalumab has demonstrated encouraging outcomes in the treatment of individuals suffering from advanced solid tumors (NCT02983578) (Reilley et al. 2018).

TGF-β promotes MDSC expansion, differentiation and immunosuppressive functions. A TGF-βR inhibitor, LY3200882, was used in a clinical study (NCT02937272) in patients with solid tumors (Yap et al. 2021). As an anti-cancer therapy, inhibition of TGF-β signaling may affect cardiac development and function, which may be a major challenge in the study. Therefore, the development of anti-TGF-β combination therapy is necessary to enhance the clinical efficacy and reduce the toxicity.

Toll-like receptor (TLR) 7/8 small molecule agonists are strong activators of mature activation in antigen-presenting cells (APCs). In a study using mice to model colon cancer, the TLR 7/8 agonist resiquimod (R848) has been shown to reduce MDSCs within tumors and in the bloodstream. In addition, it inhibits the immunosuppressive capacity of MDSCs. In another study, the combination of oxaliplatin and the Toll-like receptor agonist R848 was found to disrupt the differentiation of myeloid-derived suppressor cells (MDSCs), thereby enhancing oxaliplatin resistance in colorectal cancer patients. In addition, this combination therapy improves the anti-tumor efficacy of oxaliplatin (Liu et al. 2020).

Histone deacetylase (HDAC) inhibitors are able to impair MDSC-mediated immunosuppression. It has been shown that when the HDAC 6 inhibitor ricolinostat was used in combination with the HDAC 1 inhibitor Entinostat, both MDSC populations were completely eliminated and tumor progression was delayed in several tumor models (Luke et al. 2023). However, the HDAC 6 inhibitor ricolinostat alone only reduced M-MDSC, failed to reduce PMN-MDSC and did not reduce tumor growth in the tumor models. A similar situation occurred with Entinostat alone (Hashimoto et al. 2020). Valproic acid is an HDAC inhibitor that has been shown to reduce tumor infiltration by MDSCs, attenuate immunosuppression caused by MDSCs, and improve the efficacy of anti-PDL1 immunotherapy (Xie et al. 2020). Entinostat combination therapy has shown positive response in advanced solid tumors, as reported in a phase 2 clinical trial (NCT 01928576).

In a clinical trial (NCT02903914), it was demonstrated that small molecule inhibitors of ARG-1 can reduce levels of iNOS and COX-2, while also regulating immunosuppressive functions in MDSC. The drug combination, which included the ARG-1 inhibitor CB-1158, was proven to be both safe and effective in patients with advanced and metastatic tumors.

A recent clinical study (NCT03043313) conducted on individuals diagnosed with colorectal cancer has shown promising results in the use of tucatinib, a small molecule HER2 inhibitor, in combination with trastuzumab. and was well tolerated by the participants (Strickler et al. 2023).

During a phase 3 clinical trial (NCT00843635), tadalafil treatment was found to be well tolerated and had a positive impact on patients with HNSCC by lowering MDSC levels. It was observed that higher doses of tadalafil had no relevant immunomodulatory activity, but intermediate doses were found to be most effective.

## Targeted therapy related to colorectal cancer

### Current targeted therapies for colorectal cancer

Looking at Table 2, we can see that although some phase I and small phase II clinical trials have achieved interesting results, phase II and III trials in a wider patient population have not shown convincing efficacy, and the treatment also faces the challenge of drug-related toxicity and the emergence of drug resistance. In the meantime, researchers are exploring combinations of drugs, exploring new targets, and refining targeted therapies in the future to enhance the quality of life and prognosis for patients with bowel cancer.

**Table 2:** Current research related to targeted therapies for colorectal cancer

Drug	Drug combination	Pathway	Advantages	Limitations	Research phase	References
5-FU	Oxaliplatin, bevacizumab	Induced MDSC death	demonstrated the efficacy and feasibility of targeting the PD-1/PD-L1 or CD39/CD73 ectonucleotidase pathways for treating mCRC; demonstrated that gMDSC depletion with a better prognosis	Small sample size; uncertain whether gMDSC accumulation is an independent prognostic factor for mCRC	Clinical trial	Limagne et al. (2016a, b)
cetuximab	/	Targeted EGFR	Demonstrated efficacy and safety of cetuximab maintenance therapy	TIME trial did not reach primary endpoint	Phase 2 clinical trials	Boige et al. (2023)
Panitumumab/Bevacizumab	mFOLFOX 6	Targeted EGFR	Demonstrated longer survival in RAS wild-type patients with mFOLFOX 6 combination therapy with panitumumab	Just a randomized study, still needs repeated validation	Phase 3 clinical trials	Morano et al. (2019)
Aflibercept	FOLFIRI	Targeted VEGF	Evaluated the Efficacy, Safety and Pharmacokinetics of Abciximab combinate with FOLFIRI Therapy	Small sample size, study lacks control group	Phase 3 clinical trials	Denda et al. (2019)
Ramucirumab	FOLFOX-6	Targeted VEGFR	Demonstrated efficacy and safety of Ramucirumab therapy	Just a single-arm design, small sample size	Phase 2 clinical trials	Garcia-Carbonero et al. (2014)
Regorafenib	/	Blocked several protein kinases activity	First demonstrated small molecule multikinase inhibitor with survival benefit in mCRC	Mechanism of regorafenib in CRC needs to elucidate	Phase 3 clinical trials	Grothey et al. (2013)
Fruquintinib	/	Targeted VEGFR	Demonstrated efficacy of Furaquintinib in mCRC treatment	There are currently no proven biomarkers to predict the efficacy of Furoquinotinib, the subject population is geographically limited	Phase 3 clinical trials	Qin et al. (2021)
Famitinib	/	Inhibited receptor tyrosine kinases	Demonstrated efficacy and safety of Famitinib in mCRC treatment	Small sample size	Phase 2 clinical trials	Xu et al. (2017)
Anlotinib	/	broad spectrum of inhibitory activity on tumor angiogenesis and growth	Demonstrated similar efficacy to sunitinib in mCRC with a better safety profile for amlotinib	Small sample size, efficacy in high-risk patients has not been evaluated	Phase 2 clinical trials	Zhou et al. (2019)
Apatinib	/	Targeted VEGFR	Demonstrated the efficacy and safety of Apatinib in the treatment of mCRC	Just a single-arm design, small sample size	Phase 2 clinical trials	Chen et al. (2019)
Capecitabine	Bevacizumab	Targeted high expressed thymidine phosphorylase tumor cells	Demonstration of the efficacy and safety of capecitabine in combination with bevacizumab in the treatment of mCRC, the first and largest randomised controlled phase 3 study	Repeatability validation still required	Phase 3 clinical trials	André et al. 2023)
Pembrolizumab	/	Inhibited PD-1	Demonstrated efficacy and safety of pembrolizumab compared to standard chemotherapy in the treatment of mCRC	No overall survival reported	Phase 3 clinical trials	André et al. (2020)
Nivolumab	Regorafenib	Inhibited PD-1 mAb	Demonstrated safety and efficacy of regorafenib in combination with navulizumab in the treatment of mCRC	Just a single-arm design, the results are hypotheses, still need to be repeated for verification	Phase 2 clinical trials	Fakih et al. (2023a, b)
Ipilimumab	Regorafenib + Nivolumab	Targeted CTLA-4	Demonstrated efficacy of Ipilimumab in combination with Regorafenib + Nivolumab	Small sample size, the optimal dosage regimen still needs further evaluation	Phase 1 clinical trials	Fakih et al. (2023a, b)
Vemurafenib	/	Targeted BRAF	Demonstrated the poor efficacy of single-agent Vemurafenib in the treatment of mCRC	Small sample size	Phase 2 clinical trials	Kopetz et al. (2015)
Pertuzumab	Trastuzumab	Targeted HER2	Demonstrated efficacy of Pertuzumab in combination with trastuzumab in the treatment of CRC	Lack of randomization or control groups in the study	Phase 2 clinical trials	Meric-Bernstam et al. (2019)
/	Anti-programmed cell death protein 1	Knocked out SLC25A22, Inhibited CXCL1 production, impaired MDSC recruitment	Demonstrated the SLC25A22-asparagine-SRC/ETS2/CXCL1 metabolic circuit, demonstrated the efficacy of targeted SLC25A22 therapy in KRAS-mutant CRC	Lack of safety assessment for clinical application	Pre-clinical stage	Zhou et al. (2023)
/	Anti-programmed cell death protein 1 (BE0146; Bio-X-Cell)	Knock out METTL3, reduced MDSC accumulation	Demonstrated METTL3 promotes the m6A-BHLHE41-CXCL1/CXCR2 metabolic axis, recruits MDSCs, inhibits CD8 + T cells, promotes CRC, demonstrated the efficacy of targeted METTL3 in combination with anti-PD-1	Lack of efficacy assessment of knockdown of METTL3 alone and lack of safety assessment	Pre-clinical stage	Chen et al. (2022a, b)

*FOLFIR* 5-fluorouracil/levofolinate/irinotecan, *M CRC* metastatic colorectal cancer

### Challenges, opportunities, and possible future directions

It is undeniable that additional research is necessary in the future to tackle the challenges related to the development of MDSC in inflammatory bowel disease and its link to the initiation of colorectal cancer:

- 1. Firstly, the definition of MDSC itself is indeterminate, which hinders our study of it (Hegde et al. 2021). The definition of extant DSCs is inherently limiting. Only two categories are currently defined, M-MDSC and G-MDSC. but there are no known major regulators of MDSC properties and a range of MDSC-like phenotypes are observed in inflammation or cancer. Therefore, we need to develop a new research idea to refine the definition of MDSC.
- 2. Can MDSC be used as a biomarker of true response? It is not yet known and clinical trials are still needed to shed light on the issue. Due to the relatively small number of colitis-associated colorectal cancer-related cases, many aspects are not yet clear. As its incidence increases significantly, we should carry out large-scale, multicenter population-based cohort studies to formulate effective proactive preventive measures to reduce the occurrence of tumors; meanwhile, further studies on its cancerous mechanism will help to clarify the process of tumor development and progression, laying a theoretical basis for clinical early prevention as well as the selection of therapeutic targets.
- 3. Some other issues we need to be aware of:

Targeting molecules related to MDSCs can lead to anti-tumor effects that may be difficult to replicate in a clinical environment. Firstly, equivalence from mouse tumor models to human MDSC therapies is a challenge. Second, might there be functional differences between human and murine cell subpopulations? Human neutrophils may possess a more potent ability to kill tumor cells. Long-term inhibition of all neutrophils may be undesirable.

The activity of MDSCs may vary depending on the type of cancer and the stage of the disease. The types of MDSCs that play a major role may also be different in different cancer types. For example, PMN-MDSCs are the predominant cell population undergoing proliferation in various forms of cancer. However, in tumors like melanoma, M-MDSCs play a more critical role. Secondly, the mechanisms by which MDSCs may mediate inhibitory activity may be different at different stages of cancer. Therefore, it is important to identify the population of patients targeting MDSCs and their choice of drugs for different targets.

Moreover, different subtypes of MDSC such as PMN-MDSCs and M-MDSCs have different differentiation pathways, and different mechanisms of activity inhibition. Targeting a specific group of cells alone may not be effective in managing tumor growth. It is important to explore the combined targeting of multiple therapy approaches to effectively control tumor progression. There is a subset of patients who actually have no increase in MDSCs. Therefore, MDSC targeting may not be effective in these patients.

Therefore, the selection of therapeutic targets is important. And finding combinations of different drugs for different biological pathways may be a more effective way to target MDSCs. However, the toxicity potential of the drugs used, and the appropriate maximum clinical dose are critical issues to consider. Therefore, when selecting patients to be enrolled in the study, we need to analyze the tumor in detail, such as focusing on the detailed tumor stage and so on. At the same time, we should provide rational and individualized drug treatment plans for different categories of CAC patients. Moreover, we should focus on the development of preclinical models to achieve the transition from preclinical to clinical, and explore the reasonable combinations of treatments that can be adopted in the clinic.

## Conclusion

Colitis-associated colorectal cancer is a specific form of colorectal cancer that develops as a result of inflammatory bowel disease (IBD). MDSCs, short for myeloid-derived suppressor cells, are a group of immature myeloid cells with immunosuppressive abilities that are crucial in the progression of inflammation and cancer. There is increasing evidence suggesting that MDSCs play a crucial role in the progression of colitis-associated colorectal cancer. Under pathological states such as inflammatory microenvironment and tumor, multiple immune pathways such as STAT3, NF-κB, COX-2/PGE2 activate to recruit MDSCs to exert the corresponding immunosuppressive functions, while some inflammatory cytokines and chemokines can promote the proliferation of MDSCs and stimulate MDSCs to release reactive oxygen radicals (ROS), inducible nitric oxide synthase (iNOS), Arginase 1 (Arg-1), and other compounds, these compounds impede T cell activity and induce T cell apoptosis, and can cause intestinal cell damage by changing epithelial permeability, destroying the intestinal epithelial barrier, and mismanaging the intestinal flora, leading to heterogeneous hyperplasia of intestinal epithelial cells and proliferation of tumor cells, as well as prompting intestinal epithelial cells to undergo irreversible cancerous changes, and transforming from inflammatory bowel disease to colorectal cancer.

Immunotherapy has opened new avenues for the treatment of some malignancies. However, those used at this stage, e.g., Monoclonal antibodies targeting PD-1/PD-L1, have good efficacy only in some patients. Therefore, improving the effectiveness of immunotherapy is an important research direction. W what’s more, this paper summarizes the strategies for targeting MDSC-related therapies as well as the possible strategies for treating CAC against MDSC at this stage, to provide a reference for the discovery of more potential targets for MDSC in the future. Although there are many therapeutic targets for MDSC, the key questions of how to better improve T-cell efficacy and how to accurately combat MDSC remain to be revealed in future clinical studies. Perhaps in the near future, MDSC targeted therapy may open a new era of tumor immunotherapy.

## Data Availability

No data was used for the research described in the article.
